# The role of tumor metabolism as a driver of prostate cancer progression and lethal disease: results from a nested case-control study

**DOI:** 10.1186/s40170-016-0161-9

**Published:** 2016-12-07

**Authors:** Rachel S. Kelly, Jennifer A. Sinnott, Jennifer R. Rider, Ericka M. Ebot, Travis Gerke, Michaela Bowden, Andreas Pettersson, Massimo Loda, Howard D. Sesso, Philip W. Kantoff, Neil E. Martin, Edward L. Giovannucci, Svitlana Tyekucheva, Matthew Vander Heiden, Lorelei A. Mucci

**Affiliations:** 1 Department of Epidemiology, Harvard T.H. Chan School of Public Health, Boston, MA USA; 2 Channing Division of Network Medicine, Department of Medicine, Brigham and Women’s Hospital and Harvard Medical School, Boston, MA USA; 3 Department of Biostatistics, Harvard T.H. Chan School of Public Health, Boston, MA USA; 4 Department of Epidemiology, College of Medicine and College of Public Health and Health Professions, University of Florida, Gainesville, FL USA; 5 Center for Molecular Oncologic Pathology, Dana-Farber Cancer Institute, Boston, MA USA; 6 Clinical Epidemiology Unit, Department of Medicine Solna, Karolinska Institutet, Stockholm, Sweden; 7 Department of Pathology, Brigham and Women’s Hospital and Harvard Medical School, Boston, MA USA; 8 Division of Preventive Medicine, Brigham and Women’s Hospital and Harvard Medical School, Boston, MA USA; 9 Department of Medical Oncology, Dana-Farber Cancer Institute, Harvard Medical School, Boston, MA USA; 10 Department of Radiation Oncology, Brigham and Women’s Hospital/Dana-Farber Cancer Institute, Harvard Medical School, Boston, MA USA; 11 Department of Nutrition, Harvard T.H. Chan School of Public Health, Boston, MA USA; 12 Department of Biostatistics and Computational Biology, Dana-Farber Cancer Institute, Boston, MA USA; 13 Koch Institute for Integrative Cancer Research at Massachusetts Institute of Technology, Cambridge, MA 02139 USA; 14 Dana-Farber Cancer Institute, Boston, MA USA; 15 Broad Institute of Harvard and Massachusetts Institute of Technology, Cambridge, MA 02139 USA; 16 Channing Division of Network Medicine, 181 Longwood Avenue, Boston, MA 02115 USA

**Keywords:** Prostate cancer, mRNA expression profiling, Tumor metabolism, Metabolomic pathways, Tumorigenesis

## Abstract

**Background:**

Understanding the biologic mechanisms underlying the development of lethal prostate cancer is critical for improved therapeutic and prevention strategies. In this study we explored the role of tumor metabolism in prostate cancer progression using mRNA expression profiling of seven metabolic pathways; fatty acid metabolism, glycolysis/gluconeogenesis, oxidative phosphorylation, pentose phosphate, purine metabolism, pyrimidine metabolism and the tricarboxylic acid cycle.

**Methods:**

The study included 404 men with archival formalin-fixed, paraffin-embedded prostate tumor tissue from the prospective Health Professionals Follow-up Study and Physicians’ Health Study. Lethal cases (*n* = 113) were men who experienced a distant metastatic event or died of prostate cancer during follow-up. Non-lethal controls (*n* = 291) survived at least 8 years post-diagnosis without metastases. Of 404 men, 202 additionally had matched normal tissue (140 non-lethal, 62 lethal). Analyses compared expression levels between tumor and normal tissue, by Gleason grade and by lethal status. Secondary analyses considered the association with biomarkers of cell proliferation, apoptosis and angiogenesis.

**Results:**

Oxidative phosphorylation and pyrimidine metabolism were identified as the most dysregulated pathways in lethal tumors (*p* < 0.007), and within these pathways, a number of novel differentially expressed genes were identified including *POLR2K* and *APT6V1A*. The associations were tumor specific as there was no evidence any pathways were altered in the normal tissue of lethal compared to non-lethal cases.

**Conclusions:**

The results suggest prostate cancer progression and lethal disease are associated with alterations in key metabolic signaling pathways. Pathways supporting proliferation appeared to be of particular importance in prostate tumor aggressiveness.

**Electronic supplementary material:**

The online version of this article (doi:10.1186/s40170-016-0161-9) contains supplementary material, which is available to authorized users.

## Background

It is well known that proliferating tumor cells have different metabolic requirements from normal, differentiated cells [1]. These metabolic needs are reflected, at least in part, by a shift in metabolic phenotype including the increased conversion of glucose to lactate even when oxygen is abundant, a phenomenon termed aerobic glycolysis or the Warburg effect [2]. Rapidly replicating cancer cells require the accumulation of sufficient biomass for cell growth, and increased glucose uptake is hypothesized to support these requirements [3, 4]. Consequently, tumors exhibit altered levels of many metabolites associated with cell growth as well as energetics, stress, and tissue-specific biochemistry [5, 6]. However, what is less well understood are the differences in metabolism between tumors by their degrees of severity.

Prostate cancer represents a particularly appropriate malignancy in which to explore this question. It is the second leading cause of cancer mortality in men in many Western countries, with more than 29,000 deaths in the USA in 2014 attributable to prostate cancer [7]. Yet, at the same time, most diagnoses will not prove fatal [5]. There is a wealth of evidence supporting an important role for dysregulated metabolism in prostate cancer. One of the most consistently cited risk factors is metabolic syndrome, a collection of patho-physiological entities including visceral obesity, insulin resistance, low HDL-cholesterol, high triglycerides, elevated C-reactive protein, and low adiponectin levels [8]. Crucially, the high concentrations of immune markers and the chronic inflammation associated with this syndrome are thought to enhance tumor growth [9]. suggesting that the metabolomic signatures of prostate tumors may also reflect their aggressive potential [5]. We recently reported that the pyrimidine biosynthesis pathway, which is responsible for production of nucleic acids needed for cell replication, is enriched in higher Gleason grade tumors [10]. Here we extend these analyses to explore other relevant metabolic pathways and to consider lethal disease as an endpoint.

The objective of this study was to investigate the metabolic pathways that underlie the development and progression of prostate cancer using an integrative molecular epidemiology approach. We characterized metabolic signatures at the messenger RNA (mRNA) level in prostate tumors and adjacent benign tissue among men with prostate cancer, and compared the expression profiles across Gleason grade and cancer outcomes. The analysis was focused on genes encoding enzymes involved in metabolic pathways to further characterize the role of dysregulated metabolism as a key driver in tumor aggressiveness and prostate cancer mortality. There is compelling experimental evidence to support this hypothesis; malignant cells are known to possess unique metabolic phenotypes that differ from the healthy tissues from which they originated [1–4], yet little data to date in a population-based study of prostate cancer patients. Specifically, we will focus on seven Kyoto Encyclopaedia of Genes and Genomes (KEGG) [11] defined pathways thought to be critical in tumor cellular metabolism, particularly within the prostate: fatty acid metabolism, glycolysis/gluconeogenesis, oxidative phosphorylation, pentose phosphate, purine metabolism, pyrimidine metabolism, and the tricarboxylic acid (TCA) cycle. By deconvoluting and quantifying the contribution of each of the pathways, and by isolating key driver genes within them, these findings will provide a uniquely comprehensive understanding of the metabolic processes underlying the development of lethal prostate cancer with considerable potential for direct translation to prostate cancer patients.

## Methods

### Study population

This study was nested among men with incident prostate cancer from the prospective US Health Professionals Follow-up Study (HPFS) or Physicians’ Health Study (PHS). The HPFS [12] is a cohort study initiated in 1986 among 51,529 male health professionals aged 40 to 75 years. The PHS [13–15] was initiated as a randomized trial of aspirin and beta-carotene for the primary prevention of cardiovascular disease and cancer among 22,071 US male physicians aged 40 to 84 years.

Prostate cancer diagnosis is first reported on questionnaires and confirmed through medical record review. Clinical and pathological data including age at diagnosis, prostate-specific antigen (PSA) levels, and tumor stage are abstracted through medical record review. Post-diagnosis, men with prostate cancer are followed through questionnaires to collect information on their cancers’ clinical course, including development of metastases. Cancer-specific and all-cause mortality is ascertained through mailings and telephone calls to participants, and periodic searches of the National Death Index. A committee of physicians assigns cause of death through medical record and death certificate review. Follow-up for mortality is available through 2011 and is >98% complete.

This study includes 404 men with available archival formalin-fixed, paraffin-embedded (FFPE) prostate tumor tissue who were part of a whole genome expression profiling project of lethal prostate cancer: 150 men from PHS and 254 from HPFS. The study included lethal cases (*n* = 113) defined as men with prostate cancer who experienced a distant metastatic event or died of prostate cancer during follow-up, and non-lethal prostate cancer cases (*n* = 291) who neither died of prostate cancer nor presented any evidence of metastases during follow-up and lived at least 8 years post-diagnosis. Mean follow-up was 13.2 years (range 0.1–27.4 years). Of 404 men, 202 additionally had profiling for matched normal tissue (140 non-lethal cases, 62 lethal cases). Hematoxylin and eosin slides from all cases underwent standardized histopathologic review, including for Gleason grade and perineural invasion, by study pathologists [16].

### Tissue samples and mRNA profiling

The validated mRNA profiling methodologies were described previously [17, 18]. Briefly, RNA was extracted from two to three 0.6-mm cores within tumor regions with high cell density (>80% cellularity) and normal tissue, then amplified using the WT-Ovation FFPE SystemV2 (Nugen, San Carlos, CA). Reverse transcription was used to create a complementary DNA (cDNA)/mRNA hybrid, then the cDNA was amplified, fragmented, and labeled for hybridization to a GeneChip Human Exon 1.0 ST microarray (Affymetrics, Santa Clara, CA). The raw probe-level data was normalized through robust multichip averaging [17, 19] and probe annotation information obtained from the R package pd.hugene.1.0.st.v1 [20]. Probes not corresponding to genes were excluded, and where multiple probes corresponded to a single gene, the probe demonstrating the greatest variability was used. A total of 20,254 unique named genes were available. The genes comprising the specific metabolic pathways of interest were identified using the KEGG [11] and extracted from the whole transcriptome data.

The seven metabolic pathways are comprised of 444 unique genes. Of these, the expression profiles of 426 passed quality control criteria and were included in these analyses (Additional file 1: Table S1).

### Other tissue biomarkers

Tumor biomarkers were available for a subset of participants, and these methods have been described previously. Cellular proliferation was characterized using Ki-67 staining [21], apoptosis using TUNEL staining [21], and tumor angiogenesis using microvessel density as defined by expression of endothelial cell marker CD34 [22].

### Statistical analysis

Differences in baseline characteristics between the lethal and non-lethal cases were assessed using Student’s *t* test and the chi-squared test for continuous and categorical outcomes, respectively.

To explore the role of the metabolic pathways in disease progression, normalized expression levels were compared in tumor versus normal tissue, in lethal versus non-lethal cases, and in Gleason grade ≥8 versus 2–7 cases. Secondary analyses considered associations with markers of apoptosis, angiogenesis and cell proliferation, and the presence of perineural invasion. A variety of innovative statistical methods were integrated to capture and quantify both individual gene and pathway level effects.

Individual gene associations for each outcome were computed for the 426 genes using multivariable logistic regression models to estimate odds ratios and 95% confidence intervals. Gene expression levels were modeled as continuous independent variables. Age at diagnosis, cohort (HPFS, PHS), year of diagnosis, and body mass index (BMI) at diagnosis were included as potential confounders. For within person tumor versus normal comparisons, conditional logistic regression was used.

Pathway level associations were explored using the Global test [23], a score test designed to detect effects across many genes in a pathway. The Global test was performed by comparing, for each KEGG pathway, a logistic regression model fitted with all the genes comprising that pathway and the potential confounders to a model including only the confounders. For models with secondary biomarkers divided into quartiles, a multicategory Global test was used. For the tumor-normal comparisons, matching was dropped for these pathway tests.

We also performed Gene Set Enrichment Analysis (GSEA) [24], a competitive test in which the differential expression of genes in the pathway is compared to differential expression of genes not involved in the pathway. GSEA determines the relative importance of the explored pathways and informs on the direction of effect. For tumor-normal comparisons, the genes were ranked according to their paired *t* test statistic and the GSEA *p* values were calculated using gene permutations; for the other analyses, standard GSEA was used with *p* values calculated by permuting individuals.

Finally, a shrinkage and selection method, Least Absolute Shrinkage and Selection Operator (LASSO), was used to identify the genes contributing to pathway level associations and to determine the effect size. This approach fits a penalized regression model including all genes from each pathway as potential covariates and forces the gene expression coefficients not contributing to lethal outcome to zero so that they are removed from the model. The amount of shrinkage applied to the coefficients depends on a tuning parameter, which was chosen by using leave-one-out cross-validation to optimize the likelihood.

All analyses were conducted using the R software package.

## Results

Table 1 presents the clinical features of the lethal and non-lethal cases. Lethal cases were more likely to be older and to have a higher Gleason grade, tumor stage, and PSA level at diagnosis. They were also more likely to have a higher BMI at both baseline and diagnosis.

**Table 1:** Baseline and clinical characteristics of 404 participants with prostate cancer from the PHS and HPFS

Characteristic	Non-lethal (*n* = 291)	Lethal (*n* = 113)
Cohort, *N* (%)		
PHS	120 (41.2%)	30 (26.5%)
HPFS	171 (58.8%)	83 (73.5%)
Age at diagnosis, mean (SD)	64.9 (6.2)	67.5 (6.7)
Clinical tumor stage, *N* (%)^a^		
T1/T2 N0/Nx M0/Mx	271 (94.1%)	79 (72.5%)
T3 N0/Nx M0/Mx	16 (5.6%)	11 (10.1%)
T4/N1/M1	1 (0.3%)	19 (17.4%)
Gleason grade, *N* (%)		
2–6	56 (19.2%)	1 (0.9%)
3 + 4	126 (43.3%)	13 (11.5%)
4 + 3	67 (23.0%)	35 (31.0%)
8–10	42 (14.4%)	64 (56.6%)
PSA at diagnosis, ng/ml, *N* (%)^b^		
0–3.9	29 (10.7%)	4 (5.7%)
4–10	163 (60.1%)	35 (50.0%)
10–19.9	54 (19.9%)	15 (21.4%)
>20	25 (9.2%)	16 (22.9%)
Tissue from RP, *N* (%)	283 (97.3%)	86 (76.1%)
BMI at diagnosis, mean (SD)	25.1 (2.8)	25.9 (3.3)
BMI at baseline, mean (SD)	24.6 (2.5)	25.6 (3.2)
Matched normal tissue available	140 (48.1%)	62 (54.9)

^a^Clinical tumor stage was unknown for 3 (1%) non-lethal cases and 4 (3.5%) lethal cases

^b^PSA was unknown for 20 (6.9%) non-lethal cases and 43 (38.1%) lethal cases

### Associations of metabolic pathways with tumorigenesis

A total of 247 (58%) genes in the metabolic pathways were differentially expressed in tumor vs. normal tissue (*p ≤* 0.05) (Additional file 1: Table S2), and 118 (28%) retained significance after Bonferroni correction for multiple testing at *p* < 1.2 × 10^−4^ (0.05/426). The most significantly overexpressed genes in tumor tissue were *CANT1* (OR18.5, *p* = 5.07 × 10^−13^), *CMPK1* (OR 18.9, *p* = 2.35 × 10^−12^), and *GUCY1A3* (OR 8.0, *p* = 2.52 × 10^−12^) annotated with the purine and pyrimidine pathways; *FBP1* (OR 12.8, *p* = 2.75 × 10^−12^) and *GPI* (OR 60.7, *p* = 2.19 × 10^−12^) annotated with the pentose phosphate and glycolysis pathways. The most significantly downregulated gene in tumor tissue was *ALDH3A2* (OR 0.03, *p* = 3.23 × 10^−12^) annotated with the KEGG glycolysis and fatty acid metabolism pathways. The differences in mRNA expression between tumor versus normal tissue were similar among the lethal and non-lethal cases (results not shown).

All seven pathways showed altered expression between tumor and adjacent normal prostate tissue according to the Global test (*p* < 1 × 10^−10^) (Table 2). Using GSEA testing, pyrimidine metabolism (*p* = 0.002), purine metabolism (*p* = 0.006), and oxidative phosphorylation (*p* < 0.0001) were significant at a Bonferroni corrected threshold of *p* < 0.007 (Table 3). All pathways were upregulated in the tumor versus normal tissue.

**Table 2:** Logistic Global test *p* values for pathway level associations of metabolic pathways with tumorigenesis, Gleason grade, and lethal disease

Pathway (*n* genes)	Tumor tissue vs normal tissue (*n* = 202)	Gleason grade >8 tumors vs Gleason grade 2–7 tumors (*n* = 404;>8 = 106/2–7 = 298)	Lethal tumors vs. non-lethal tumors (*n* = 404; L = 113/NL = 291)
Fatty acid metabolism (*n* = 39)	<1.0 × 10^−10^	0.03	1.4 × 10^−4^
Glycolysis/gluconeogenesis (*n =* 62)	<1.0 × 10^−10^	1.4 × 10^−5^	<1.0 × 10^−10^
Pentose phosphate (*n =* 27)	<1.0 × 10^−10^	3.5 × 10^−3^	8.9 × 10^−5^
Purine metabolism (*n =* 157)	<1.0 × 10^−10^	8.8 × 10^−6^	1.2 × 10^−8^
Pyrimidine metabolism (*n =* 96)	<1.0 × 10^−10^	7.6 × 10^−5^	1.9 × 10^−7^
Oxidative phosphorylation (*n =* 123)	<1.0 × 10^−10^	8.3 × 10^−5^	3.5 × 10^−6^
TCA (*n =* 30)	<1.0 × 10^−10^	0.78	0.21

All tests compare the null model including only age at diagnosis, cohort (PHS, HPFS), year of diagnosis, and BMI at diagnosis to the full model which also includes the genes in the listed pathway. Matching information is dropped for the tumor vs. normal comparison

*NL* non-lethal, *L* lethal

**Table 3:** *p* value and direction of upregulation of the seven metabolic pathways for tumorigenesis, Gleason grade, and lethal prostate cancer, according to the Gene Set Enrichment Analysis

Pathway (*n* genes)	Tumor tissue vs normal tissue (*n* = 202)		Gleason grade >8 tumors vs Gleason grade 2–7 tumors (*n* = 404; >8 = 106/2–7 = 298)		Lethal tumors vs. non-lethal tumors (*n* = 404; L = 113/NL = 291)	
	*p* value	Expression in tumor tissue	*p* value	Expression in high Gleason grade (>8) tumors	*p* value	Expression in lethal tumors
Fatty acid metabolism (*n* = 39)	0.70	↑	0.37	↓	0.14	↓
Glycolysis/gluconeogenesis (*n* = 62)	0.18	↑	0.75	↑	0.44	↓
Pentose phosphate (*n* = 27)	0.03	↑	0.21	↑	0.73	↑
Purine metabolism (*n* = 157)	6.0 × 10^−3^	↑	0.03	↑	0.07	↑
Pyrimidine metabolism (*n* = 96)	2.0 × 10^−3^	↑	3.0 × 10^−3^	↑	6.0 × 10^−3^	↑
Oxidative phosphorylation (*n* = 123)	<0.0001	↑	4.0 × 10^−3^	↑	6.0 × 10^−3^	↑
TCA (*n* = 30)	0.05	↑	0.64	↑	0.76	↑

For the tumor vs. normal comparison matching is maintained and the GSEA-Preranked procedure is used with gene-based permutation *p* values; for the other two comparisons, standard GSEA is used with sample-based permutation *p* values

LASSO regression was then used to identify the genes contributing to the observed pathway level associations. Most of the significant associations for each pathway under the individual gene tests were also identified by the LASSO regression, and several additional genes were suggested to be of importance (Additional file 1: Table S2). These may represent genes that are important in a pathway context, when all other potentially interacting genes are taken into account.

### Associations of metabolic pathways with Gleason grade

We next compared tumor expression levels of the metabolic genes in Gleason 2–7 tumors vs. Gleason ≥8 tumors (Additional file 1: Figure S1). A total of 101 (24%) genes were associated with Gleason grade (*p ≤* 0.05), of which six genes retained significance after Bonferroni correction. *HPRT1* (purine metabolism pathway, OR 5.2, *p* = 1.93 × 10^−6^), *RRM2* (purine and pyrimidine metabolism pathways, OR = 3.8, *p* = 6.50 × 10^−6^), *PDE4D* (purine metabolism pathway, OR 0.3, *p* = 1.22 × 10^−5^), and *NDUFC2* (oxidative phosphorylation pathway, OR = 3.6, *p* = 1.64 × 10^−5^) ranked as the most significant (Additional file 1: Table S3). Results were similar when Gleason 7 cancers (3 + 4 and 4 + 3) were excluded and Gleason 2–6 vs. Gleason 8–10 tumors were compared (results not shown).

At a pathway level, purine metabolism (*p* = 8.8 × 10^−6^), glycolysis/gluconeogenesis (*p* = 1.37 × 10^−5^), pyrimidine metabolism (*p* = 7.56 × 10^−5^), and oxidative phosphorylation (*p =* 8.3 × 10^−5^) were significant under the Global test (Table 2). Pyrimidine metabolism (*p =* 0.003) and oxidative phosphorylation (*p =* 0.004) were also significant under GSEA (Table 3). Most of the significant genes identified using logistic regression also ranked under the LASSO model, but novel genes were also identified.

### Associations of metabolic pathways with lethal status

**Fig. 1:**
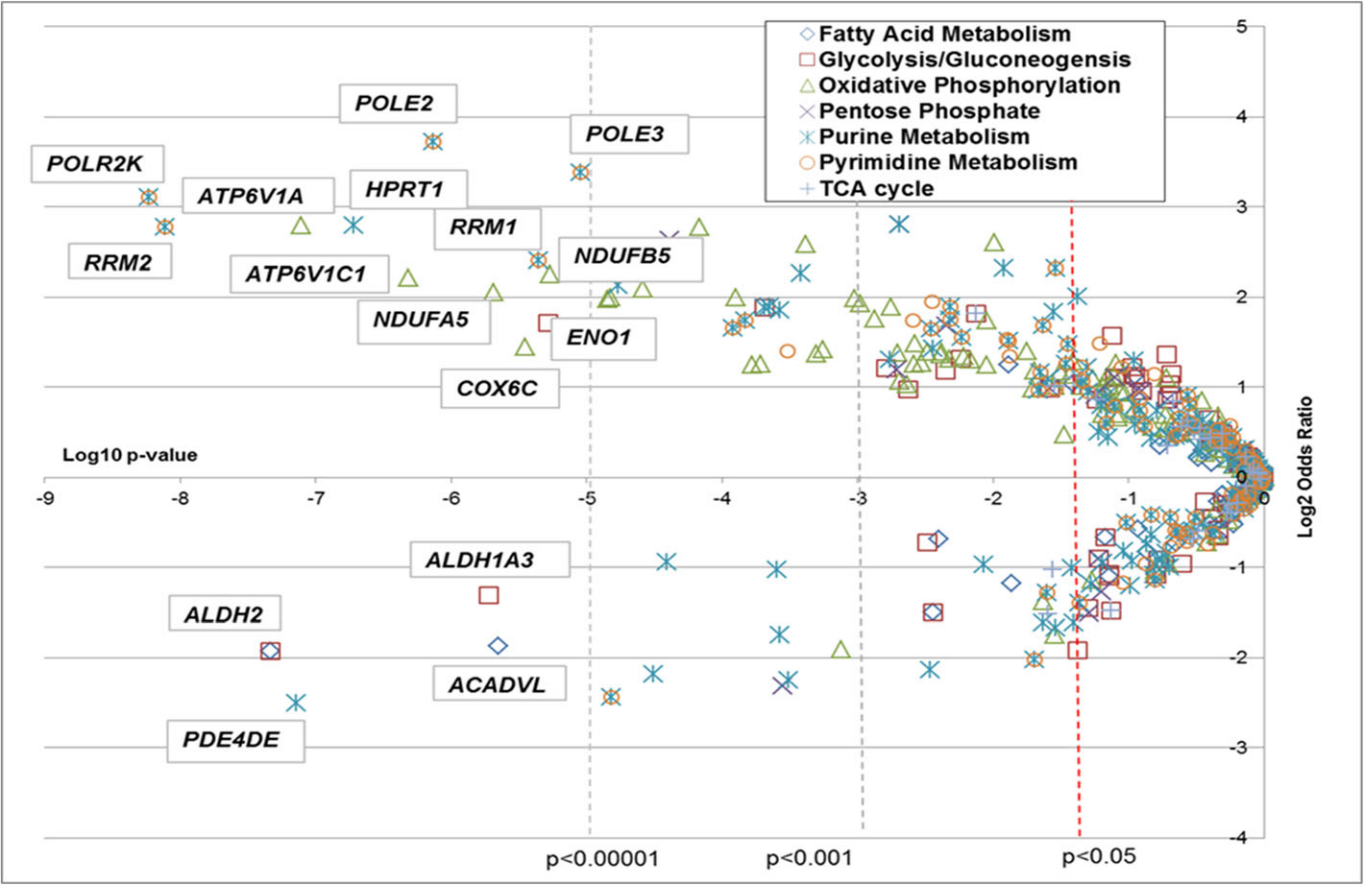
Strength and significance of individual gene associations with lethal prostate cancer among seven metabolic pathways. Log odds ratios computed using a logistic regression model adjusting for age at diagnosis, cohort (HPFS, PHS), year of diagnosis, and BMI at diagnosis

Tumor expression of 124 genes was associated with lethal vs. non-lethal outcome (*p ≤* 0.05). The associated odds ratios and *p* values are described in Fig. 1. Three-quarters (*n* = 98, 76%) of these genes were more highly expressed in the lethal cases (Additional file 1: Table S4). Twenty-six genes retained significance after Bonferroni correction. *POLR2K*, part of the purine and pyrimidine metabolism pathways, ranked as the top differentially expressed gene (OR 8.6, *p =* 5.84 × 10^−9^). *RRM2* (OR 6.8 *p =* 7.72 × 10^−9^), *POLE2* (OR 13.2, *p =* 7.36 × 10^−7^) (both associated with purine and pyrimidine metabolism), and *ATP6V1A* (OR 6.9, *p =* 7.81 × 10^−8^) (oxidative phosphorylation) were also expressed to higher levels in lethal tumors, while *ALDH2* (OR 0.3, *p =* 4.65 × 10^−8^ (associated with fatty acid metabolism and glycolysis), *PDE4D* (OR 0.2, *p =* 7.14 × 10^−8^, purine metabolism), and *ALDH1A3* (OR 0.4, *p =* 1.89 × 10^−6^, glycolysis) ranked as the most significantly downregulated genes. The associations with lethal disease were attenuated but still significant when adjusting for Gleason grade (Additional file 1: Figure S2).

All but the TCA cycle pathway were significantly associated with lethal disease by the Global test at the Bonferroni corrected threshold (Table 2). Most significant associations retained significance or borderline significance when additionally adjusting for Gleason grade (Additional file 1: Table S5).

To determine whether the metabolic pathway gene expression changes may be influenced by gene expression changes in the surrounding tissue, expression levels were compared in the morphologically normal prostate tissue from lethal cases (*n* = 62) with the normal tissue from non-lethal (*n* = 140) cases. None of the pathways were identified as significant according to the Global test (*p* > 0.05 for all pathways), and thus the relationship between lethal disease and altered metabolic gene expression appears to be a tumor-specific effect.

Intriguingly, the strongest associations were apparent in the overweight/obese men (Additional file 1: Table S5). There was some evidence that the associations were restricted to low-stage (T1/T2 N0/Mx) tumors, although the stage-stratified analyses were limited by small numbers. Again the pathways of pyrimidine metabolism (*p* = 0.006) and oxidative phosphorylation (*p* = 0.006) were significant under GSEA analysis (Table 3), with both pathways upregulated in the lethal compared to non-lethal tumors. The drivers of these pathway associations were explored using LASSO regression (Additional file 1: Table S4), and the findings suggested a role for both the top hits from the logistic regression model as well as a number of additional genes that may be acting jointly on risk.

### Associations with additional secondary outcomes

We explored the association between expression of the metabolic pathways with biomarkers of cell proliferation, apoptosis, angiogenesis, and perineural invasion. The strongest associations of the pathways were with cell proliferation (*p* < 0.007) (Table 4). Conversely, there was no Bonferroni corrected significant association with apoptosis nor angiogenesis. Extent of perineural invasion was significantly associated with fatty acid metabolism (*p* = 1.1 × 10^−6^), glycolysis/gluconeogenesis (*p* = 1.2 × 10^−4^), purine metabolism (*p* = 1.9 × 10^−5^), and pyrimidine metabolism (*p* = 3.5 × 10^−4^).

**Table 4:** Multicategory Global test *p* value for pathway level associations of tumor expression of the metabolic pathways with histologic and molecular features of prostate cancer

Pathway (*n* genes)	Ki-67 quartiles (*n* = 314 men)	Apoptosis quartiles (*n* = 255 men)	Microvessel density quartiles^a^(*n* = 174 men)	Perineural invasion^a^(*n* = 132 men)
Fatty acid metabolism (*n* = 39)	1.8 × 10^−4^	0.20	0.01	1.1 × 10^−6^
Glycolysis/gluconeogenesis (*n* = 62)	9.4 × 10^−9^	0.19	0.06	1.2 × 10^−4^
Pentose phosphate (*n* = 27)	4.0 × 10^−6^	0.20	0.14	0.04
Purine metabolism (*n* = 157)	4.1 × 10^−6^	0.42	0.09	1.9 × 10^−5^
Pyrimidine metabolism (*n* = 96)	2.8 × 10^−6^	0.64	0.17	3.5 × 10^−4^
Oxidative phosphorylation (*n* = 123)	3.1 × 10^−8^	0.09	2.0 × 10^−3^	0.02
TCA (*n* = 30)	1.1 × 10^−7^	0.30	0.04	0.37

All tests compare the null model including only age at diagnosis, cohort (PHS, HPFS) if data was available for both, year of diagnosis, and BMI at diagnosis to the full model which also includes the genes in the listed pathway

^a^Data was only available for HPFS so cohort was excluded from these models

## Discussion

Cancer cells share a metabolic requirement to support inappropriate cell proliferation and maintain growth [1]; however, the ways in which these requirements are fulfilled differ across tumor types. In particular, the healthy prostate is known to exhibit a unique metabolism, enabling the production of the components of prostatic fluid: PSA, spermine, myso-inositol, and citrate. This is disrupted in neoplastic cells due to the loss of ability to accumulate zinc and subsequently to accumulate citrate. Therefore prostate tumors display unique metabolomic alterations [25, 26]. These metabolic alterations may vary according to tumor aggressiveness; however, the majority of studies to date exploring this question have focused on cell lines or model organism. None have focused on prostate cancer within a patient cohort [2, 27]. The results of this novel study provide human-based evidence that key metabolic pathways are relevant to prostate cancer lethality. Therefore, the findings have the potential for forward translation into the identification of therapeutic targets and the development of biomarkers that better define tumors by their degree of aggressiveness.

Consistent with previous literature [3], in this study, we demonstrate alterations in key metabolic genes and pathways within the prostate during tumorigenesis. Crucially, we additionally offer evidence that these changes differ in cancer progression by histologic grade and by lethal outcome. Changes in prostate tumor gene expression were dominated by increased expression in the lethal or high-grade cancers, which may suggest that this subset of aggressive prostate tumors have increased metabolic activity. This is supported by studies in other tumors reporting increased nutrient uptake by FDG-PET is associated with more aggressive cancer [28].

Among the top differentially expressed genes, many were common between the tumorigenesis, lethal and Gleason analyses, while others appeared to be specific to disease aggressiveness. A number of the top genes identified in this study have previously been implicated in prostate cancer development and progression, Gleason grade, metastases and biochemical recurrence; including *CANT1* [29], *FBP1* [30], *RRM2* [31], *POLE2* [32], *PDE4D* [33], and *ALDH1A3* [34]. This supports the validity of our findings and strengthens the evidence for the role of these genes by demonstrating their differential expression levels in a human study.

Additionally, we report a number of novel genes with biological plausibility; this is the first study to report on the association between *GPI* and *ALDH3A2* with prostate cancer in a human population. Mammalian GPI has been demonstrated to function as a tumor-secreted cytokine and angiogenic factor, while cells with high ALDH activity have been shown to display metastasis-initiating behavior [35]. A further ALDH isoform, *ALDH2*, has previously been associated with the progression of benign prostatic hyperplasia [36], but this study represents the first to specifically link it with lethal prostate cancer*.* Similarly, we report novel associations between Gleason grade with *HPRT1* and *NDUFC2.* NADH dehydrogenase has been repeatedly implicated in prostate cancer risk [37], and both these genes play vital roles in the generation of the purine nucleotides necessary to support proliferation.

On a pathway level, the predominance of gene expression changes associated with glycolysis and oxidative phosphorylation pathways provides support for a shift in metabolism toward increased aerobic glycolysis in tumors with lethal potential [3]. Changes in gene expression in the other investigated pathways may help dividing cells fulfill proliferation requirements that extend beyond ATP production [2]. The increased expression of genes encoding enzymes in the purine and pyrimidine metabolism pathways in lethal relative to non-lethal tumors may support increased DNA replication and cell proliferation in these tumors, while changes in the pentose phosphate pathway and fatty acid metabolism genes may reflect the increased need of lethal tumors for nucleotides, amino acids, and lipids [4, 27]. This is further supported by the association of these pathways with biomarkers of cell proliferation and is in agreement with the enrichment of genes of pyrimidine metabolism in high Gleason grade tumors [10]. Interestingly, there was no difference in metabolic enzyme expression of the seven KEGG pathways observed in the adjacent normal tissue from patients who developed lethal prostate cancer compared to those who did not develop lethal disease, supporting a tumor-specific effect.

There are potential limitations to this study. A significantly larger proportion of the non-lethal cases had biopsy tissue available from transurethral resection of the prostate as opposed to radical prostatectomy (RP), and our recent study suggests that the expression levels of some genes differs as a function of zone of origin [10]. However, sensitivity analyses restricting to the RP cases produced comparable results. A hypothesis-led approach was taken for the selection of metabolic pathways, although it is possible that other metabolic pathways not considered in these analyses may be exerting an effect. In this study, we were unable to measure metabolite concentrations and inferred the metabolic state based on expression of metabolic pathways. However, the regulatory mechanisms determining the relationship between gene expression and metabolite levels are complex and not yet fully understood [38]. Transcript levels and metabolite abundances do not overlap directly with the underlying biochemical pathways [39]. The correlations between transcripts and metabolites may be influenced by time lags, reaction kinetics, network effects, feedback reactions, or noise. Furthermore, post-translational modification can have substantial impacts on metabolite levels [38, 39].

Nevertheless, transcriptional regulation is known to play an important role in the control of metabolism [40], and thus can be considered a good proxy with which to explore our pathways of interest. This study represents one of the largest of its kind to date and, to the best of our knowledge, is the first to use in silico analysis of gene expression profiling to discover new hypotheses that may underlie lethal prostate cancer link. We focus on important but as yet underexplored hypotheses, based on strong biological rational, to provide an increased mechanistic understanding of lethal prostate cancer. The nesting within two population-based studies with a rich variety of epidemiological, anthropometric, and lifestyle data allows us to control for possible confounding factors and evaluate effect modifiers. Furthermore, the methodologies utilized in this study have been previously validated within our included cohorts. The availability of matched normal tissue allowed us to consider the potential role of the tumor microenvironment on our findings, and the possibility of a field effect, representing a further strength of this study.

## Conclusions

In conclusion, prostate tumorigenesis is associated with dysregulation of key metabolic signaling pathways. Within tumor tissue, we offer evidence of specific alterations that differ by Gleason grade and lethal outcome and identify novel genes that may play an important role. Our findings add further support to the hypotheses that metabolic alterations in aggressive tumors may extend beyond an increase in aerobic glycolysis and encompass changes in gene expression for enzymes involved in other pathways supporting proliferation [2, 4, 41]. A thorough understanding of the metabolic phenotypes of aggressive tumors may help us to better define the mechanisms underlying lethal prostate cancer and illuminate novel therapeutic targets, to improve outcomes for those men at greatest risk of prostate cancer death [2, 4, 42].

## Additional file

Additional file 1: Table S1. The number of available genes from the seven selected KEGG-defined metabolic pathways and the number of common genes between the pathways, after the exclusion of genes that failed quality control. **Table S2** Genes associated with tumorigenesis in 202 prostate cancer cases with tumor and normal tissue available according to a conditional logistic regression model and a Least Absolute Shrinkage and Selection Operator (LASSO) regression model. **Table S3** Genes associated with Gleason grade (Grade 2–7 versus grade 8) in 404 prostate cancer cases according to a logistic regression model and a Least Absolute Shrinkage and Selection Operator (LASSO) regression model. **Table S4** Genes associated with lethal disease in 404 prostate cancer cases (291 non-lethal and 113 lethal) cases according to a logistic regression model and a Least Absolute Shrinkage and Selection Operator (LASSO) regression model. **Table S5** Global test *p* value for pathway level associations with lethal prostate cancer exploring potential effect modifiers. **Figure S1** Strength (log odds ratios) and significance (log *p* values) of individual gene associations with Gleason grade ≥8 (*n* = 106) versus Gleason grade 2–7 (*n* = 298) tumors across the seven pathways. **Figure S2** Strength (log odds ratios) and significance (log *p* values) of individual gene associations with lethal prostate cancer among the seven metabolic pathways after additional adjustment for Gleason grade. (DOCX 337 kb)

## Abbreviations

ATP: Adenosine triphosphate
BMI: TUNEL staining body mass index
FDG-PET: Fludeoxyglucose - positron emission tomography
FFPE: Formalin-fixed, paraffin-embedded
GSEA: Gene Set Enrichment Analysis
HPFS: Health Professionals Follow-up Study
IRB: Institutional review boards
KEGG: Kyoto Encyclopaedia of Genes and Genomes
LASSO: Least Absolute Shrinkage and Selection Operator
PHS: Physicians’ Health Study
PSA: Prostate-specific antigen
RP: Radical prostatectomy
TCA: Tricarboxylic acid
