# Supplementary material for: The role of tumor metabolism as a driver of prostate cancer progression and lethal disease: results from a nested case-control study
Source: Cancer Metab. 2016 Dec 7;4:22. doi: 10.1186/s40170-016-0161-9 (PMC5142400; doi:10.1186/s40170-016-0161-9)
Supplement: Additional file 1: Table S1. — The number of available genes from the seven selected KEGG-defined metabolic pathways and the number of common genes between the pathways, after the exclusion of genes that failed quality control. Table S2 Genes associated with tumorigenesis in 202 prostate cancer cases with tumor and normal tissue available according to a conditional logistic regression model and a Least Absolute Shrinkage and Selection Operator (LASSO) regression model. Table S3 Genes associated with Gleason grade (Grade 2–7 versus grade 8) in 404 prostate cancer cases according to a logistic regression model and a Least Absolute Shrinkage and Selection Operator (LASSO) regression model. Table S4 Genes associated with lethal disease in 404 prostate cancer cases (291 non-lethal and 113 lethal) cases according to a logistic regression model and a Least Absolute Shrinkage and Selection Operator (LASSO) regression model. Table S5 Global test p value for pathway level associations with lethal prostate cancer exploring potential effect modifiers. Figure S1 Strength (log odds ratios) and significance (log p values) of individual gene associations with Gleason grade ≥8 (n = 106) versus Gleason grade 2–7 (n = 298) tumors across the seven pathways. Figure S2 Strength (log odds ratios) and significance (log p values) of individual gene associations with lethal prostate cancer among the seven metabolic pathways after additional adjustment for Gleason grade. (DOCX 337 kb) [file 40170_2016_161_MOESM1_ESM.docx]

**Additional file**

**Table S1 The number of available genes from the seven selected KEGG-defined metabolic pathways and the number of common genes between the pathways, after the exclusion of genes that failed quality control**

**Table S2 Genes associated with tumorigenesis in 202 prostate cancer cases with tumor and normal tissue available according to a conditional logistic regression model and a Least Absolute Shrinkage and Selection Operator (LASSO) regression model**

**Table S3 Genes associated with Gleason grade (Grade 2-7 versus grade 8) in 404 prostate cancer cases according to a logistic regression model and a Least Absolute Shrinkage and Selection Operator (LASSO) regression model**

**Table S4 Genes associated with lethal disease in 404 prostate cancer cases (291 non-lethal and 113 lethal) cases according to a logistic regression model and a Least Absolute Shrinkage and Selection Operator (LASSO) regression model**

**Table S5 Global test p-value for pathway-level associations with lethal prostate cancer exploring potential effect modifiers**

**Figure S1 Strength (log odds ratios) and significance (log p-values) of individual gene associations with Gleason grade ≥8 (n=106) versus Gleason grade 2-7 (n=298) tumors across the seven pathways**

**Figure S2 Strength (log odds ratios) and significance (log p-values) of individual gene associations with lethal prostate cancer among the seven metabolic pathways after additional adjustment for Gleason grade**

**Table S1 The number of available genes from the seven selected KEGG-defined metabolic pathways and the number of common genes between the pathways, after the exclusion of genes that failed quality control**

|  | **Fatty Acid metabolism (n=39)** | **Glycolysis/Gluconeogenesis (n=62)** | **Pentose Phosphate (n=27)** | **Purine Metabolism (n=157)** | **Pyrimidine Metabolism (n=96)** | **Oxidative phosphorylation (n=123)** | **TCA (n=30)** |
| --- | --- | --- | --- | --- | --- | --- | --- |
| **Fatty acid metabolism (n=39)** | **39** |  |  |  |  |  |  |
| **Glycolysis/Gluconeogenesis (n=62)** | 12 | **62** |  |  |  |  |  |
| **Pentose phosphate (n=27)** | 0 | 11 | **27** |  |  |  |  |
| **Purine metabolism (n=157)** | 0 | 2 | 3 | **157** |  |  |  |
| **Pyrimidine metabolism (n=96)** | 0 | 0 | 0 | 69 | **96** |  |  |
| **Oxidative phosphorylation (n=123)** | 0 | 0 | 0 | 0 | 0 | **123** |  |
| **TCA (n=30)** | 0 | 7 | 0 | 0 | 0 | 4 | **30** |

*Corresponding to 426 unique genes*

**Table S2 Genes associated with tumorigenesis in 202 prostate cancer cases with tumor and normal tissue available according to a conditional logistic regression model and a Least Absolute Shrinkage and Selection Operator (LASSO) regression model**

| **Gene** | **Logistic Regression Model** | | **LASSO Model** | **Gene** | **Logistic Regression Model** | | | **LASSO Model** |
| --- | --- | --- | --- | --- | --- | --- | --- | --- |
|  | **OR** | **p-value** | **Coefficient** |  | **OR** | **p-value** | | **Coefficient** |
| ***Fatty acid metabolism*** | | | | ***Glycolysis/ Gluconeogenesis*** | | | | |
| *ALDH3A2* | 0.03 | 3.2E-12 | -2.38 | *GPI* | 60.67 | | 2.2E-12 | 1.12 |
| *ALDH2* | 0.12 | 4.1E-11 | -0.73 | *FBP1* | 12.79 | | 2.8E-12 | 0.83 |
| *ECHS1* | 35.37 | 2.3E-10 | 1.35 | *ALDH3A2* | 0.03 | | 3.2E-12 | -1.61 |
| *ACSL3* | 3.17 | 2.7E-09 | 0.33 | *LDHA* | 11.29 | | 1.8E-11 | 0.71 |
| *ACSL5* | 3.48 | 7.7E-08 | 0.64 | *ALDH2* | 0.12 | | 4.1E-11 | -0.80 |
| *ACSL4* | 0.17 | 3.8E-07 |  | *LDHB* | 0.16 | | 1.1E-10 | -0.43 |
| *ACADSB* | 3.01 | 2.0E-06 | 0.39 | *ACSS1* | 35.45 | | 2.5E-10 |  |
| *CYP4A11* | 0.35 | 8.1E-05 |  | *ALDH1A3* | 7.35 | | 2.8E-10 | 0.44 |
| *ACSL1* | 1.96 | 9.4E-05 |  | *AKR1A1* | 20.58 | | 4.1E-10 | 0.09 |
| *ADH4* | 0.26 | 1.0E-04 | -0.41 | *ENO1* | 7.31 | | 2.4E-08 | 0.04 |
| *HADHB* | 4.15 | 1.4E-04 |  | *ALDH3B2* | 5.85 | | 8.1E-07 |  |
| *ACADL* | 0.57 | 1.5E-04 | -0.37 | *GAPDH* | 4.48 | | 1.6E-06 | 0.05 |
| *ADH5* | 0.39 | 2.0E-04 | -0.18 | *PGM2* | 4.04 | | 7.3E-06 |  |
| *ACADVL* | 0.36 | 0.001 |  | *ADH4* | 0.26 | | 1.0E-04 |  |
| *EHHADH* | 2.43 | 0.001 | 0.83 | *HK2* | 2.11 | | 1.6E-04 |  |
| *ACADM* | 2.33 | 0.003 | 0.12 | *ALDH3A1* | 0.12 | | 1.7E-04 |  |
| *ACOX1* | 2.77 | 0.003 | 0.14 | *PGM1* | 0.19 | | 2.0E-04 |  |
| *CPT2* | 2.53 | 0.005 | 0.19 | *ADH5* | 0.39 | | 2.0E-04 |  |
| *ALDH9A1* | 0.36 | 0.007 | -0.64 | *PGAM1* | 0.16 | | 0.001 |  |
| *HADH* | 2.64 | 0.008 | 0.57 | *PGAM4* | 0.17 | | 0.001 |  |
| *ACAA2* | 0.45 | 0.016 |  | *ENO2* | 0.27 | | 0.001 |  |
| *ADH1A* | 0.54 | 0.020 |  | *ACSS2* | 0.28 | | 0.001 |  |
| *ACAT1* | 2.00 | 0.024 | 0.12 | *PGK1* | 3.19 | | 0.001 |  |
| *ACOX3* | 2.06 | 0.026 | 0.35 | *PFKP* | 0.40 | | 0.001 |  |
| *HADHA* | 1.88 | 0.033 | 0.17 | *PFKL* | 4.01 | | 0.001 |  |
| ***Pentose phosphate*** | | | | *TPI1* | 2.23 | | 0.001 |  |
| *GPI* | 60.67 | 2.2E-12 | 1.62 | *HK1* | 0.23 | | 0.002 |  |
| *FBP1* | 12.79 | 2.8E-12 | 1.11 | *ALDH9A1* | 0.36 | | 0.007 |  |
| *TALDO1* | 18.26 | 4.3E-08 |  | *BPGM* | 2.89 | | 0.016 |  |
| *TKT* | 6.72 | 4.4E-08 | 0.59 | *PGAM2* | 0.42 | | 0.016 |  |
| *PRPS1* | 3.98 | 2.0E-07 | 0.63 | *ADH1A* | 0.54 | | 0.020 |  |
| *PGM2* | 4.04 | 7.3E-06 | 0.31 | *ALDOC* | 0.46 | | 0.032 |  |
| *PGD* | 3.15 | 7.6E-06 |  | *DLAT* | 2.51 | | 0.035 |  |
| *DERA* | 0.17 | 8.4E-06 | -0.61 | *PDHB* | 1.88 | | 0.036 |  |
| *PGM1* | 0.19 | 2.0E-04 | -0.87 |  |  | |  |  |
| *PFKP* | 0.40 | 0.001 | -0.49 |  |  | |  |  |
| *PFKL* | 4.01 | 0.001 |  |  |  | |  |  |
| *H6PD* | 0.33 | 0.001 |  |  |  | |  |  |
| *TKTL2* | 0.33 | 0.016 |  |  |  | |  |  |
| *RPIA* | 2.31 | 0.029 |  |  |  | |  |  |
| *ALDOC* | 0.46 | 0.032 |  |  |  | |  |  |

**Table S2 Genes associated with tumorigenesis in 202 prostate cancer cases with tumor and normal tissue available according to a conditional logistic regression model and a Least Absolute Shrinkage and Selection Operator (LASSO) regression model *continued***

| **Gene** | **Logistic Regression Model** | | **LASSO Model** | | **Gene** | **Logistic Regression Model** | | **LASSO Model** | **Gene** | **Logistic Regression Model** | | **LASSO Model** |
| --- | --- | --- | --- | --- | --- | --- | --- | --- | --- | --- | --- | --- |
|  | **OR** | **p-value** | **Coefficient** | |  | **OR** | **p-value** | **Coefficient** |  | **OR** | **p-value** | **Coefficient** |
| ***Oxidative phosphorylation*** | | | | | ***Oxidative phosphorylation*** | | | | ***Oxidative phosphorylation*** | | | |
| *ATP6V1G1* | 23.65 | 3.6E-12 | | 0.94 | *ATP5H* | 2.85 | 0.001 |  | *COX11* |  |  | -0.04 |
| *COX5A* | 22.11 | 1.5E-11 | | 0.15 | *ATP5A1* | 3.15 | 0.001 |  | *COX2* |  |  | 0.96 |
| *NDUFV2* | 18.01 | 1.7E-11 | | 1.15 | *ATP5C1* | 2.61 | 0.001 |  | *COX6A2* |  |  | 0.60 |
| *ATP5B* | 19.79 | 4.6E-11 | | 0.46 | *COX6B2* | 0.14 | 0.001 |  | *COX6C* |  |  | -0.23 |
| *UQCRH* | 4.27 | 9.2E-10 | | 0.48 | *ATP6AP1* | 2.92 | 0.001 | -0.12 | *ND2* |  |  | 0.35 |
| *NDUFB10* | 14.47 | 9.9E-10 | | 0.83 | *ATP6V1F* | 3.36 | 0.002 |  | *NDUFA6* |  |  | -0.40 |
| *COX7A1* | 0.11 | 3.6E-09 | | -0.29 | *NDUFAB1* | 2.06 | 0.002 | -0.15 | *NDUFB6* |  |  | -0.74 |
| *ATP6V0B* | 10.56 | 3.7E-08 | |  | *NDUFB2* | 3.79 | 0.002 |  | *NDUFB8* |  |  | -0.47 |
| *PPA1* | 4.21 | 7.6E-08 | | 0.48 | *COX7A2* | 2.50 | 0.002 |  | *NDUFS2* |  |  | 0.54 |
| *PPA2* | 11.93 | 1.4E-07 | |  | *NDUFB1* | 2.81 | 0.002 |  |  |  |  |  |
| *NDUFA2* | 6.17 | 5.0E-07 | |  | *UQCRFS1* | 1.94 | 0.003 | 0.23 |  |  |  |  |
| *ATP5G1* | 7.16 | 1.4E-06 | | 0.54 | *COX7B* | 1.58 | 0.003 | 0.19 |  |  |  |  |
| *TCIRG1* | 0.13 | 1.4E-06 | | -0.24 | *ATP6V1D* | 2.13 | 0.004 |  |  |  |  |  |
| *NDUFS6* | 9.22 | 1.8E-06 | |  | *UQCRC2* | 3.33 | 0.004 |  |  |  |  |  |
| *ND6* | 0.08 | 2.4E-06 | | -0.40 | *ATP6V1B2* | 2.50 | 0.004 | 0.04 |  |  |  |  |
| *NDUFS7* | 0.13 | 2.5E-06 | |  | *NDUFA9* | 2.61 | 0.005 | 0.53 |  |  |  |  |
| *ATP5E* | 4.88 | 3.3E-06 | | 0.55 | *ATP6V0E1* | 2.96 | 0.005 |  |  |  |  |  |
| *ATP6V1G3* | 0.05 | 3.9E-06 | |  | *ATP6V0A4* | 0.33 | 0.006 | -0.19 |  |  |  |  |
| *NDUFS1* | 5.84 | 4.4E-06 | | 0.38 | *COX4I2* | 0.34 | 0.009 |  |  |  |  |  |
| *NDUFB4* | 5.00 | 7.4E-06 | |  | *NDUFA5* | 1.75 | 0.01 |  |  |  |  |  |
| *NDUFC2* | 4.79 | 7.4E-06 | |  | *ATP6V1B1* | 0.30 | 0.01 |  |  |  |  |  |
| *ATP6V1C1* | 3.96 | 1.3E-05 | | 0.12 | *ATP5I* | 2.91 | 0.012 |  |  |  |  |  |
| *CYC1* | 4.67 | 1.6E-05 | | 0.10 | *UQCR11* | 2.21 | 0.012 |  |  |  |  |  |
| *ATP6V0E2* | 11.33 | 2.5E-05 | |  | *NDUFS4* | 0.44 | 0.014 | -0.64 |  |  |  |  |
| *NDUFB9* | 4.12 | 2.5E-05 | |  | *NDUFS3* | 2.92 | 0.017 |  |  |  |  |  |
| *ND5* | 0.15 | 3.0E-05 | | -1.31 | *SDHD* | 0.74 | 0.018 | -0.26 |  |  |  |  |
| *COX7A2L* | 3.81 | 3.8E-05 | |  | *ATP5D* | 3.07 | 0.021 | 0.12 |  |  |  |  |
| *NDUFA1* | 2.85 | 4.4E-05 | |  | *NDUFC1* | 2.46 | 0.024 | -0.07 |  |  |  |  |
| *NDUFA8* | 3.56 | 5.3E-05 | |  | *UQCRC1* | 2.60 | 0.028 |  |  |  |  |  |
| *ATP5G2* | 7.49 | 5.9E-05 | |  | *ATP4B* | 0.31 | 0.029 |  |  |  |  |  |
| *NDUFB5* | 3.60 | 6.6E-05 | | 0.28 | *COX6B1* | 1.99 | 0.029 |  |  |  |  |  |
| *ATP6V1A* | 3.45 | 7.9E-05 | |  | *SDHC* | 0.56 | 0.036 |  |  |  |  |  |
| *COX17* | 2.80 | 9.0E-05 | | 0.05 | *NDUFS5* | 0.53 | 0.038 | -0.75 |  |  |  |  |
| *ATP5J2* | 2.80 | 1.1E-04 | | -0.32 | *NDUFA7* | 1.91 | 0.041 | -0.31 |  |  |  |  |
| *COX6A1* | 4.75 | 3.2E-04 | | -0.28 | *ATP5L* | 1.86 | 0.050 |  |  |  |  |  |
| *COX8A* | 4.09 | 3.3E-04 | |  | *ATP12A* |  |  | 0.11 |  |  |  |  |
| *ATP6V0D2* | 0.16 | 3.5E-04 | | -0.15 | *ATP5O* |  |  | -0.13 |  |  |  |  |
| *UQCRQ* | 2.61 | 3.6E-04 | |  | *ATP6V0A2* |  |  | -0.13 |  |  |  |  |
| *SDHB* | 2.87 | 4.4E-04 | |  | *ATP6V1E1* |  |  | -0.45 |  |  |  |  |
| *UQCR10* | 3.38 | 0.001 | | -1.8E-03 | *ATP6V1H* |  |  | -0.03 |  |  |  |  |

**Table S2 Genes associated with tumorigenesis in 202 prostate cancer cases with tumor and normal tissue available according to a conditional logistic regression model and a Least Absolute Shrinkage and Selection Operator (LASSO) regression model *continued***

| **Gene** | **Logistic Regression Model** | | | **LASSO Model** | **Gene** | **Logistic Regression Model** | | | | **LASSO Model** | **Gene** | **Logistic Regression Model** | | | **LASSO Model** |
| --- | --- | --- | --- | --- | --- | --- | --- | --- | --- | --- | --- | --- | --- | --- | --- |
|  | **OR** | | **p-value** | **Coef.** |  | **OR** | | **p-value** | | **Coef.** |  | **OR** | **p-value** | **Coef.** | |
| ***Purine metabolism*** | | | | | ***Purine metabolism*** | | | | | | ***Purine metabolism*** | | | | |
| *CANT1* | 18.48 | 5.1E-13 | | 0.69 | *PDE4B* | | 0.25 | | 3.9E-05 |  | *PDE4C* | 0.30 | 0.015 | |  |
| *GUCY1A3* | 7.98 | 2.5E-12 | | 0.91 | *ADCY4* | | 0.23 | | 4.2E-05 |  | *NME7* | 0.40 | 0.018 | |  |
| *IMPDH2* | 17.63 | 3.1E-12 | | 0.62 | *POLE3* | | 8.24 | | 4.4E-05 |  | *ZNRD1* | 1.61 | 0.025 | |  |
| *ADCY5* | 0.05 | 6.2E-12 | | -0.61 | *ENPP3* | | 6.53 | | 4.5E-05 |  | *ADA* | 0.38 | 0.025 | |  |
| *ENTPD5* | 4.55 | 8.8E-12 | | 0.32 | *NME4* | | 5.73 | | 4.9E-05 |  | *AK4* | 1.60 | 0.027 | |  |
| *NME1* | 15.06 | 9.0E-12 | | 0.02 | *ADCY6* | | 5.93 | | 5.7E-05 |  | *POLR2B* | 2.00 | 0.033 | |  |
| *ADSL* | 12.63 | 3.5E-10 | | 0.17 | *NUDT2* | | 4.86 | | 6.7E-05 |  | *NME5* | 0.46 | 0.034 | |  |
| *PAICS* | 8.89 | 1.2E-09 | |  | *GMPR* | | 0.48 | | 7.9E-05 | -0.43 | *ADCY10* | 3.76 | 0.044 | |  |
| *NPR2* | 0.04 | 1.3E-09 | |  | *ADCY3* | | 0.28 | | 8.1E-05 |  | *PRIM2* | 0.70 | 0.046 | | -0.22 |
| *POLR1B* | 26.19 | 1.5E-09 | |  | *ADCY9* | | 0.22 | | 1.3E-04 |  | *POLE* | 2.60 | 0.048 | |  |
| *POLR2H* | 6.33 | 4.8E-09 | | 0.73 | *POLR2J3* | | 0.42 | | 1.7E-04 | -0.10 | *POLR2F* | 2.07 | 0.050 | |  |
| *POLD2* | 4.99 | 6.2E-09 | | 0.05 | *ADSSL1* | | 0.12 | | 1.9E-04 |  |  |  |  | |  |
| *PDE5A* | 0.37 | 1.4E-08 | | -0.03 | *PAPSS2* | | 0.37 | | 2.0E-04 |  |  |  |  | |  |
| *ENTPD6* | 9.48 | 2.4E-08 | |  | *ENPP1* | | 2.89 | | 2.9E-04 | 0.11 |  |  |  | |  |
| *NT5E* | 0.12 | 4.9E-08 | |  | *HPRT1* | | 3.98 | | 3.1E-04 |  |  |  |  | |  |
| *PDE1C* | 0.17 | 5.8E-08 | |  | *POLR2J2* | | 0.15 | | 3.8E-04 |  |  |  |  | |  |
| *PDE4A* | 0.07 | 1.2E-07 | |  | *NT5C2* | | 2.45 | | 0.001 |  |  |  |  | |  |
| *GART* | 13.72 | 1.4E-07 | |  | *AK5* | | 2.73 | | 0.001 |  |  |  |  | |  |
| *PDE3B* | 3.09 | 1.6E-07 | | 0.11 | *PPAT* | | 4.09 | | 0.001 |  |  |  |  | |  |
| *PDE11A* | 0.31 | 1.7E-07 | | -0.33 | *PDE2A* | | 0.26 | | 0.001 |  |  |  |  | |  |
| *PRPS1* | 3.98 | 2.0E-07 | | 0.48 | *GUK1* | | 3.88 | | 0.001 |  |  |  |  | |  |
| *ITPA* | 11.14 | 2.1E-07 | |  | *AK1* | | 0.26 | | 0.001 |  |  |  |  | |  |
| *PDE7B* | 0.19 | 4.4E-07 | |  | *GUCY2C* | | 0.22 | | 0.002 |  |  |  |  | |  |
| *POLR1A* | 8.14 | 6.9E-07 | |  | *FHIT* | | 2.91 | | 0.002 |  |  |  |  | |  |
| *ENTPD3* | 0.19 | 8.0E-07 | | -0.12 | *GMPS* | | 2.68 | | 0.002 |  |  |  |  | |  |
| *AK2* | 4.24 | 1.1E-06 | | 0.02 | *NUDT5* | | 2.30 | | 0.003 |  |  |  |  | |  |
| *PNPT1* | 4.07 | 1.3E-06 | |  | *PFAS* | | 3.91 | | 0.003 |  |  |  |  | |  |
| *POLR2D* | 12.94 | 1.3E-06 | |  | *POLR3A* | | 3.07 | | 0.004 |  |  |  |  | |  |
| *PDE4D* | 0.20 | 1.9E-06 | |  | *PDE6H* | | 0.16 | | 0.004 |  |  |  |  | |  |
| *ADSS* | 5.55 | 2.3E-06 | |  | *POLR1C* | | 2.97 | | 0.005 |  |  |  |  | |  |
| *PDE9A* | 2.79 | 2.4E-06 | |  | *PDE1A* | | 2.73 | | 0.006 |  |  |  |  | |  |
| *POLR1D* | 5.17 | 2.4E-06 | |  | *GUCY2D* | | 0.20 | | 0.006 |  |  |  |  | |  |
| *PAPSS1* | 4.99 | 4.1E-06 | |  | *NT5C* | | 0.27 | | 0.007 |  |  |  |  | |  |
| *POLR3GL* | 0.25 | 5.5E-06 | | -0.01 | *IMPDH1* | | 5.09 | | 0.007 |  |  |  |  | |  |
| *PNP* | 4.31 | 1.2E-05 | | 0.27 | *APRT* | | 3.10 | | 0.009 |  |  |  |  | |  |
| *RRM2* | 4.60 | 2.0E-05 | |  | *ADCY2* | | 0.50 | | 0.010 | -0.28 |  |  |  | |  |
| *NUDT9* | 4.94 | 2.1E-05 | |  | *POLR2I* | | 2.44 | | 0.011 |  |  |  |  | |  |
| *ENTPD1* | 0.26 | 2.6E-05 | |  | *DGUOK* | | 2.24 | | 0.012 |  |  |  |  | |  |
| *GUCY1B3* | 3.86 | 2.7E-05 | |  | *PDE6B* | | 0.34 | | 0.012 |  |  |  |  | |  |
| *PDE8B* | 0.54 | 3.3E-05 | | -0.75 | *ATIC* | | 2.10 | | 0.013 |  |  |  |  | |  |

**Table S2 Genes associated with tumorigenesis in 202 prostate cancer cases with tumor and normal tissue available according to a conditional logistic regression model and a Least Absolute Shrinkage and Selection Operator (LASSO) regression model *continued***

| **Gene** | **Logistic Regression Model** | | **LASSO Model** | **Gene** | **Logistic Regression Model** | | | | **LASSO Model** |
| --- | --- | --- | --- | --- | --- | --- | --- | --- | --- |
|  | **OR** | **p-value** | **Coef.** |  | **OR** | **p-value** | | | **Coef.** |
| **Pyrimidine metabolism** | | | | **Pyrimidine metabolism** | | | | | |
| *CANT1* | 18.48 | 5.1E-13 | 0.90 | *DHODH* | 2.44 | | | 0.032 |  |
| *CMPK1* | 18.91 | 2.4E-12 | 0.96 | *POLR2B* | 2.00 | | | 0.033 |  |
| *ENTPD5* | 4.55 | 8.8E-12 | 0.34 | *NME5* | 0.46 | | | 0.034 |  |
| *NME1* | 15.06 | 9.0E-12 | 0.56 | *PRIM2* | 0.70 | | | 0.046 | -0.41 |
| *POLR1B* | 26.19 | 1.5E-09 | 0.59 | *POLE* | 2.60 | | | 0.048 |  |
| *POLR2H* | 6.33 | 4.8E-09 | 1.08 | *POLR2F* | 2.07 | | | 0.050 |  |
| *POLD2* | 4.99 | 6.2E-09 | 0.16 | *CTPS* |  | | |  | 0.06 |
| *UCK2* | 13.54 | 9.4E-09 |  | *POLR2C* |  | | |  | -0.02 |
| *ENTPD6* | 9.48 | 2.4E-08 |  | *RRM1* |  | | |  | -0.16 |
| *NT5E* | 0.12 | 4.9E-08 |  | **TCA cycle** | | | | | |
| *ITPA* | 11.14 | 2.2E-07 | 0.26 | *ACLY* | 7.20 | | 1.2E-11 | | 0.86 |
| *DPYS* | 0.08 | 4.8E-07 | -0.01 | *MDH2* | 20.50 | | 1.8E-11 | | 1.58 |
| *POLR1A* | 8.14 | 6.9E-07 |  | *FH* | 7.06 | | 8.2E-07 | | 1.14 |
| *ENTPD3* | 0.19 | 8.0E-07 | -0.39 | *CS* | 7.51 | | 1.6E-06 | | 0.61 |
| *PNPT1* | 4.07 | 1.3E-06 |  | *IDH3A* | 0.16 | | 5.0E-05 | | -0.58 |
| *POLR2D* | 12.94 | 1.3E-06 |  | *ACO1* | 0.30 | | 1.7E-04 | | -0.71 |
| *POLR1D* | 5.17 | 2.4E-06 | 0.14 | *SDHB* | 2.87 | | 4.4E-04 | | 0.12 |
| *POLR3GL* | 0.25 | 5.5E-06 | -0.47 | *IDH1* | 1.98 | | 0.001 | | -0.33 |
| *DCTD* | 5.97 | 1.1E-05 |  | *IDH3G* | 3.21 | | 0.007 | |  |
| *PNP* | 4.31 | 1.2E-05 | 0.54 | *MDH1* | 2.14 | | 0.012 | |  |
| *RRM2* | 4.60 | 2.0E-05 | 0.27 | *ACO2* | 2.72 | | 0.014 | |  |
| *UMPS* | 6.92 | 2.5E-05 |  | *SDHD* | 0.74 | | 0.018 | | -0.36 |
| *ENTPD1* | 0.26 | 2.6E-05 |  | *DLAT* | 2.51 | | 0.035 | |  |
| *POLE3* | 8.24 | 4.4E-05 |  | *SDHC* | 0.56 | | 0.036 | | -0.16 |
| *NME4* | 5.73 | 4.9E-05 |  | *PDHB* | 1.88 | | 0.036 | |  |
| *NUDT2* | 4.86 | 6.7E-05 |  | *IDH2* |  | |  | | -0.40 |
| *POLR2J3* | 0.42 | 1.7E-04 | -0.17 | *IDH3B* |  | |  | | -0.37 |
| *DPYD* | 0.37 | 3.3E-04 |  | *OGDH* |  | |  | | -0.11 |
| *POLR2J2* | 0.15 | 3.8E-04 |  | *PCK1* |  | |  | | 0.10 |
| *NT5C2* | 2.45 | 0.001 |  | *PCK2* |  | |  | | -0.17 |
| *TXNRD2* | 3.49 | 0.001 |  |  |  | |  | |  |
| *UPP2* | 0.15 | 0.003 |  |  |  | |  | |  |
| *POLR3A* | 3.07 | 0.004 |  |  |  | |  | |  |
| *CTPS2* | 2.76 | 0.004 |  |  |  | |  | |  |
| *POLR1C* | 2.97 | 0.005 |  |  |  | |  | |  |
| *CMPK2* | 0.19 | 0.005 |  |  |  | |  | |  |
| *NT5C* | 0.27 | 0.007 |  |  |  | |  | |  |
| *POLR2I* | 2.44 | 0.011 |  |  |  | |  | |  |
| *NME7* | 0.40 | 0.018 |  |  |  | |  | |  |
| *ZNRD1* | 1.61 | 0.025 |  |  |  | |  | |  |

**Table S3 Genes associated with Gleason grade (Grade 2-7 versus grade 8) in 404 prostate cancer cases according to a logistic regression model and a Least Absolute Shrinkage and Selection Operator (LASSO) regression model**

| ***Gene*** | **Logistic Regression Model** | | **LASSO Model** | ***Gene*** | **Logistic Regression Model** | | **LASSO Model** | ***Gene*** | **Logistic Regression Model** | | **LASSO Model** |
| --- | --- | --- | --- | --- | --- | --- | --- | --- | --- | --- | --- |
|  | **OR** | **p-value** | **Coef.** |  | **OR** | **p-value** | **Coef.** |  | **OR** | **p-value** | **Coef.** |
| **Fatty acid metabolism** | | | | **Oxidative phosphorylation** | | | | **Oxidative phosphorylation** | | | |
| *ACADVL* | 0.39 | 2.1E-04 | -0.398 | *COX7A1* | 0.33 | 0.003 | -0.492 | *COX5B* |  |  | -0.019 |
| *ALDH3A2* | 0.37 | 3.5E-03 |  | *NDUFB5* | 2.43 | 0.004 |  | *COX7B* |  |  | -0.168 |
| *HADHB* | 2.39 | 0.011 |  | *COX5A* | 2.23 | 0.005 | 0.024 | *ND2* |  |  | -0.159 |
| *ACSL1* | 1.52 | 0.016 | 0.114 | *CYC1* | 2.59 | 0.005 |  | *NDUFA11* |  |  | -0.099 |
| *CYP4A11* | 0.53 | 0.042 |  | *NDUFB1* | 2.60 | 0.006 |  | *NDUFA4L2* |  |  | 0.364 |
| *ACADL* |  |  | -0.024 | *ATP5E* | 2.49 | 0.006 | 0.087 | *NDUFA8* |  |  | -0.228 |
| **Glycolysis/Gluconeogenesis** | | | | *ATP6V1H* | 2.74 | 0.006 |  | *NDUFA9* |  |  | -0.121 |
| *ENO1* | 2.28 | 4.3E-04 | 0.241 | *ATP6V1A* | 2.32 | 0.008 |  | *NDUFS2* |  |  | -0.129 |
| *ALDH1A3* | 0.54 | 4.6E-04 | -0.551 | *ATP6V0B* | 2.77 | 0.008 |  | *NDUFV2* |  |  | -0.356 |
| *GAPDH* | 2.23 | 0.002 | 0.147 | *COX8A* | 2.42 | 0.008 |  | *NDUFV3* |  |  | -0.539 |
| *TPI1* | 2.31 | 0.003 | 0.178 | *NDUFB6* | 2.24 | 0.009 |  | *SDHA* |  |  | 0.132 |
| *PGM1* | 0.20 | 0.003 |  | *NDUFS5* | 2.32 | 0.01 | 0.489 | *SDHC* |  |  | -0.018 |
| *ALDH3A2* | 0.37 | 0.004 |  | *NDUFB9* | 2.12 | 0.011 |  | *SDHD* |  |  | -0.03 |
| *PKM2* | 2.50 | 0.005 | 0.147 | *COX6A1* | 2.54 | 0.011 |  | *UQCR11* |  |  | -0.036 |
| *LDHB* | 0.66 | 0.010 | -0.417 | *ATP6V1B2* | 2.26 | 0.015 | 0.32 | *UQCRC2* |  |  | -0.307 |
| *PGAM4* | 3.55 | 0.016 |  | *ATP5L* | 2.10 | 0.019 |  |  |  |  |  |
| *GPI* | 1.99 | 0.020 |  | *ATP5C1* | 2.10 | 0.019 |  |  |  |  |  |
| *LDHA* | 1.55 | 0.034 |  | *ATP5O* | 2.08 | 0.023 |  |  |  |  |  |
| *HK1* | 0.36 | 0.038 |  | *NDUFA5* | 1.83 | 0.026 |  |  |  |  |  |
| *PGM2* | 1.72 | 0.044 | 0.068 | *ATP6V1D* | 1.84 | 0.027 |  |  |  |  |  |
| *PGAM1* | 3.05 | 0.047 |  | *NDUFB4* | 1.80 | 0.033 |  |  |  |  |  |
| *PGK1* | 1.84 | 0.048 |  | *ATP6V0E1* | 2.16 | 0.035 |  |  |  |  |  |
| **Oxidative phosphorylation** | | | | *ATP5B* | 1.95 | 0.036 |  |  |  |  |  |
| *NDUFC2* | 3.63 | 1.6E-05 | 0.387 | *NDUFA2* | 1.90 | 0.039 | -0.284 |  |  |  |  |
| *ATP6V1C1* | 3.22 | 2.6E-05 | 0.231 | *ND6* | 0.55 | 0.043 | -0.308 |  |  |  |  |
| *COX6C* | 2.22 | 7.0E-05 | 0.407 | *ATP4A* |  |  | 0.202 |  |  |  |  |
| *ATP6V0C* | 6.44 | 1.7E-04 | 0.667 | *ATP5A1* |  |  | -1.105 |  |  |  |  |
| *NDUFA1* | 2.44 | 2.1E-04 | 0.455 | *ATP5F1* |  |  | -0.513 |  |  |  |  |
| *ATP5J2* | 2.70 | 2.2E-04 | 0.567 | *ATP5G2* |  |  | -0.266 |  |  |  |  |
| *NDUFAB1* | 2.26 | 3.2E-04 | 0.494 | *ATP5I* |  |  | -0.366 |  |  |  |  |
| *NDUFC1* | 4.57 | 4.6E-04 | 0.476 | *ATP5J* |  |  | -0.19 |  |  |  |  |
| *UQCRFS1* | 2.53 | 6.2E-04 | 0.52 | *ATP6V0A1* |  |  | 0.421 |  |  |  |  |
| *NDUFB3* | 2.76 | 8.8E-04 | 0.271 | *ATP6V0A2* |  |  | -0.557 |  |  |  |  |
| *NDUFB2* | 4.33 | 1.3E-03 | 0.34 | *ATP6V0D1* |  |  | -0.297 |  |  |  |  |
| *UQCRH* | 2.03 | 1.4E-03 | 0.149 | *ATP6V1G1* |  |  | -0.155 |  |  |  |  |
| *PPA2* | 3.50 | 0.002 |  | *ATP6V1G2* |  |  | 0.068 |  |  |  |  |
| *ATP6V1E2* | 0.22 | 0.002 | -0.516 | *COX11* |  |  | 0.018 |  |  |  |  |
| *NDUFV1* | 3.52 | 0.003 | 1.476 | *COX15* |  |  | -0.074 |  |  |  |  |
| *COX7A2* | 2.27 | 0.003 |  | *COX2* |  |  | -0.096 |  |  |  |  |
| *ATP5G1* | 2.91 | 0.003 | 0.363 | *COX3* |  |  | -0.311 |  |  |  |  |

**Table S3 Genes associated with Gleason grade (Grade 2-7 versus grade 8) in 404 prostate cancer cases according to a logistic regression model and a Least Absolute Shrinkage and Selection Operator (LASSO) regression model *continued***

| **Gene** | **Logistic Regression Model** | | **LASSO Model** | ***Gene*** | **Logistic Regression Model** | | | **LASSO Model** |
| --- | --- | --- | --- | --- | --- | --- | --- | --- |
|  | **OR** | **p-value** | **Coef.** |  | **OR** | | **p-value** | **Coef.** |
| **Pentose phosphate** | | | | **Purine metabolism** | | | | |
| *PGM1* | 0.2 | 0.003 |  | *POLR2H* | 2.95 | 1.7E-04 | | 0.387 |
| *PRPS1* | 1.88 | 0.008 | 0.174 | *POLE3* | 5.58 | 3.8E-04 | |  |
| *TALDO1* | 2.66 | 0.017 |  | *PDE5A* | 0.61 | 5.6E-04 | | -0.395 |
| *GPI* | 1.99 | 0.020 |  | *POLR2K* | 2.84 | 6.2E-04 | | 0.135 |
| *RPE* | 2.42 | 0.024 |  | *POLR2A* | 0.29 | 6.8E-04 | | -0.548 |
| *PGM2* | 1.72 | 0.044 |  | *GMPS* | 2.76 | 1.4E-03 | |  |
| **Pyrimidine metabolism** | | | | *POLD2* | 2.30 | 1.5E-03 | | 0.475 |
| *RRM2* | 3.84 | 6.5E-06 | 0.567 | *PDE7B* | 0.29 | 0.002 | | -0.082 |
| *DCK* | 4.97 | 1.2E-04 | 0.464 | *ADCY5* | 0.36 | 0.002 | |  |
| *POLR2H* | 2.95 | 1.7E-04 | 0.396 | *RRM1* | 2.61 | 0.004 | |  |
| *POLE3* | 5.58 | 3.8E-04 |  | *PKM2* | 2.50 | 0.005 | | 0.288 |
| *POLR2K* | 2.84 | 6.2E-04 | 0.335 | *POLA1* | 3.57 | 0.005 | | 0.015 |
| *POLR2A* | 0.29 | 6.8E-04 | -0.571 | *POLE2* | 3.68 | 0.006 | |  |
| *POLD2* | 2.3 | 1.5E-03 | 0.515 | *GMPR* | 0.61 | 0.006 | |  |
| *DHODH* | 0.23 | 0.002 | -0.252 | *GART* | 3.10 | 0.006 | |  |
| *RRM1* | 2.61 | 0.004 |  | *PDE10A* | 1.74 | 0.007 | | 0.186 |
| *POLA1* | 3.57 | 0.005 |  | *PRPS1* | 1.88 | 0.008 | | 0.433 |
| *POLE2* | 3.68 | 0.006 |  | *ATIC* | 2.38 | 0.009 | |  |
| *TXNRD1* | 2.89 | 0.006 |  | *AK5* | 1.79 | 0.009 | | 0.182 |
| *TK1* | 4.38 | 0.007 |  | *POLR3K* | 3.54 | 0.011 | |  |
| *POLR3K* | 3.54 | 0.011 |  | *ENTPD1* | 2.26 | 0.016 | | 0.513 |
| *ENTPD1* | 2.26 | 0.016 | 0.344 | *NT5C* | 0.24 | 0.017 | |  |
| *NT5C* | 0.24 | 0.017 |  | *PDE2A* | 0.28 | 0.018 | |  |
| *TYMS* | 2.06 | 0.019 | 0.133 | *POLE4* | 1.92 | 0.020 | |  |
| *POLE4* | 1.92 | 0.02 | 0.069 | *POLR2B* | 2.36 | 0.022 | |  |
| *POLR2B* | 2.36 | 0.022 |  | *ADCY1* | 0.35 | 0.027 | |  |
| *NT5M* | 3.74 | 0.041 |  | *NT5M* | 3.74 | 0.040 | |  |
| *PNPT1* | 1.81 | 0.042 |  | *PNPT1* | 1.81 | 0.042 | |  |
| *POLR2I* | 2.14 | 0.048 |  | *PDE1A* | 1.58 | 0.045 | |  |
| *NT5E* | 0.47 | 0.049 |  | *POLR2I* | 2.14 | 0.048 | |  |
| *CANT1* |  |  | -0.283 | *NT5E* | 0.47 | 0.049 | |  |
| *DPYD* |  |  | 0.015 | *AK4* |  |  | | -0.184 |
| *ENTPD5* |  |  | -0.089 | *C17orf48* |  |  | | -0.155 |
| *PNP* |  |  | 0.008 | *CANT1* |  |  | | -0.278 |
| *UPRT* |  |  | 0.087 | *ENTPD5* |  |  | | -0.261 |
| ***Purine metabolism*** | | | | *GUCY1A3* |  |  | | -0.118 |
| *HPRT1* | 5.24 | 1.90E-06 | 0.252 | *PDE3A* |  |  | | 0.18 |
| *RRM2* | 3.84 | 6.50E-06 | 0.3 | *PDE3B* |  |  | | -0.062 |
| *PDE4D* | 0.28 | 1.20E-05 | -0.468 | *POLR3GL* |  |  | | 0.241 |
| *DCK* | 4.97 | 1.20E-04 | 0.343 |  |  |  | |  |

**Table S4 Genes associated with lethal disease in 404 prostate cancer cases (291 non-lethal and 113 lethal) cases according to a logistic regression model and a Least Absolute Shrinkage and Selection Operator (LASSO) regression model**

| ***Gene*** | **Logistic Regression Model** | | **LASSO Model** | ***Gene*** | **Logistic Regression Model** | | **LASSO Model** | ***Gene*** | **Logistic Regression Model** | | | | | **LASSO Model** | |
| --- | --- | --- | --- | --- | --- | --- | --- | --- | --- | --- | --- | --- | --- | --- | --- |
|  |  |  |  |  |  |  |  |  |  |  |  |  |  |  |  |
|  | **OR** | **p-value** | **Coef.** |  | **OR** | **p-value** | **Coef.** |  | **OR** | | **p-value** | | | **Coef.** | |
| ***Fatty acid metabolism*** | | | | ***Purine metabolism*** | | | | ***Purine metabolism*** | | | | | | | |
| *ALDH2* | 0.29 | 4.03E-06 | -0.67 | *POLR2K* | 5.61 | 1.45E-05 | 0.79 | *ENTPD1* | |  | |  | | | 0.32 |
| *ACADVL* | 0.36 | 4.69E-04 | -0.52 | *RRM2* | 4.92 | 2.07E-05 | 0.3 | *ENTPD5* | |  | |  | | | -0.14 |
| *ACAT1* | 2.53 | 2.14E-02 |  | *POLE2* | 11.87 | 4.31E-05 | 0.15 | *ITPA* | |  | |  | | | -0.31 |
| *ACOX3* | 0.44 | 2.76E-02 |  | *C17orf48* | 0.27 | 5.21E-04 | -0.83 | *NT5C2* | |  | |  | | | -0.09 |
| *ACADL* |  |  | -0.07 | *PNPT1* | 3.56 | 5.81E-04 | 0.42 | *PDE10A* | |  | |  | | | -0.14 |
| *ACSL1* |  |  | 0.03 | *PRUNE* | 4.19 | 5.81E-04 | 0.55 | *PDE3A* | |  | |  | | | 0.4 |
| *CPT1A* |  |  | 0.13 | *RRM1* | 3.65 | 9.14E-04 |  | *PDE8A* | |  | |  | | | 0 |
| ***Glycolysis/Gluconeogenesis*** | | | | *HPRT1* | 3.77 | 1.09E-03 |  | *PFAS* | |  | |  | | | -0.08 |
| *ALDH2* | 0.29 | 4.03E-06 | -0.59 | *PDE4D* | 0.32 | 1.25E-03 | -0.87 | *PRPS1* | |  | |  | | | 0.09 |
| *ENO1* | 2.74 | 6.47E-04 | 0.45 | *POLE3* | 6.08 | 1.56E-03 |  | *PRPS2* | |  | |  | | | -0.12 |
| *ALDH1A3* | 0.5 | 8.47E-04 | -0.7 | *GMPS* | 3.27 | 1.91E-03 | 0.36 | ***TCA cycle*** | | | | | | | |
| *PKM2* | 2.85 | 8.82E-03 | 0.54 | *POLR2A* | 0.27 | 2.04E-03 | -0.69 | *DLAT* | | 3.55 | | 0.018 | 0.14 | | |
| *LDHA* | 1.85 | 1.54E-02 |  | *PDE11A* | 0.42 | 2.92E-03 | -0.14 | *ACLY* | |  | |  | -0.16 | | |
| *DLAT* | 3.55 | 1.85E-02 |  | *ATIC* | 3.15 | 3.26E-03 | 0.09 | *ACO1* | |  | |  | -0.05 | | |
| *GAPDH* | 1.83 | 4.66E-02 |  | *PDE6A* | 8.65 | 3.81E-03 |  | *DLD* | |  | |  | 0.47 | | |
| *PGK1* | 2.03 | 4.72E-02 |  | *POLR2E* | 0.2 | 4.81E-03 | -0.48 | *FH* | |  | |  | 0.14 | | |
| *LDHB* |  |  | -0.46 | *GART* | 4.07 | 5.80E-03 |  | *IDH1* | |  | |  | 0.1 | | |
| *PFKP* |  |  | -0.04 | *POLR3F* | 3.83 | 6.34E-03 |  | *IDH2* | |  | |  | -0.62 | | |
| *TPI1* |  |  | 0.1 | *PKM2* | 2.85 | 8.82E-03 | 1.11 | *IDH3A* | |  | |  | -0.63 | | |
| ***Pentose phosphate*** | | | | *GUK1* | 0.23 | 1.20E-02 | -0.42 | *SDHA* | |  | |  | 0.13 | | |
| *TALDO1* | 5.31 | 9.80E-04 | 1.22 | *PAICS* | 2.19 | 1.63E-02 |  | *SDHB* | |  | |  | 0.32 | | |
| *H6PD* | 0.27 | 7.05E-03 | -0.85 | *ADCY5* | 0.38 | 1.76E-02 |  |  | |  | |  |  | | |
| *PRPS1L1* | 5.37 | 1.86E-02 | 1.15 | *PRPS1L1* | 5.37 | 1.86E-02 | 0.13 |  | |  | |  |  | | |
| *PGD* | 1.97 | 2.20E-02 | 0.42 | *ADCY2* | 0.43 | 1.97E-02 | -0.39 |  | |  | |  |  | | |
| *TKTL2* | 0.28 | 2.84E-02 | -0.72 | *POLR2H* | 2.16 | 2.31E-02 | 0.26 |  | |  | |  |  | | |
| *ALDOA* |  |  | 0.33 | *PDE7B* | 0.35 | 2.56E-02 |  |  | |  | |  |  | | |
| *ALDOC* |  |  | 0.55 | *AMPD3* | 2.42 | 2.63E-02 |  |  | |  | |  |  | | |
| *FBP1* |  |  | -0.34 | *POLR1A* | 2.62 | 2.91E-02 |  |  | |  | |  |  | | |
| *GPI* |  |  | 0.14 | *ADSS* | 2.31 | 2.96E-02 |  |  | |  | |  |  | | |
| *PFKL* |  |  | 0.16 | *POLR2B* | 2.61 | 3.21E-02 |  |  | |  | |  |  | | |
| *PFKP* |  |  | -0.61 | *NT5C1B* | 5.94 | 3.22E-02 |  |  | |  | |  |  | | |
| *PGLS* |  |  | -0.23 | *GMPR* | 0.64 | 3.65E-02 |  |  | |  | |  |  | | |
| *PGM2* |  |  | -0.19 | *GUCY1A3* | 1.49 | 3.91E-02 | -0.08 |  | |  | |  |  | | |
| *PRPS2* |  |  | -0.44 | *PDE5A* | 0.72 | 4.91E-02 | -0.56 |  | |  | |  |  | | |
| *RPE* |  |  | 0.57 | *CANT1* |  |  | -0.14 |  | |  | |  |  | | |
| *TKT* |  |  | 0.14 | *ENPP3* |  |  | -0.2 |  | |  | |  |  | | |

**Table S4 Genes associated with lethal disease in 404 prostate cancer cases (291 non-lethal and 113 lethal) cases according to a logistic regression model and a LASSO regression model *cont.***

| ***Gene*** | **Logistic Regression Model** | | | **LASSO Model** | ***Gene*** | **Logistic Regression Model** | | | | | **LASSO Model** |
| --- | --- | --- | --- | --- | --- | --- | --- | --- | --- | --- | --- |
|  |  |  |  |  |  |  |  |  |  |  |  |
|  | **OR** | | **p-value** | **Coef.** |  | **OR** | | | **p-value** | | **Coef.** |
| ***Pyrimidine metabolism*** | | | | | ***Oxidative phosphorylation*** | | | | | | |
| *POLR2K* | 5.61 | 1.45E-05 | | 1.09 | *ATP6V1A* | | 4.83 | 3.91E-05 | | 0.7 | |
| *RRM2* | 4.92 | 2.07E-05 | | 0.76 | *NDUFA5* | | 3.71 | 7.29E-05 | | 0.22 | |
| *POLE2* | 11.87 | 4.31E-05 | |  | *COX6C* | | 2.14 | 1.26E-03 | | 0.35 | |
| *PNPT1* | 3.56 | 5.81E-04 | | 0.09 | *ATP5L* | | 3.28 | 1.33E-03 | |  | |
| *RRM1* | 3.65 | 9.14E-04 | | 0.20 | *NDUFB5* | | 3.3 | 1.44E-03 | | 0.07 | |
| *CMPK1* | 2.63 | 1.36E-03 | |  | *ATP6V1C1* | | 2.88 | 1.59E-03 | | 0.36 | |
| *POLE3* | 6.08 | 1.56E-03 | |  | *NDUFB3* | | 3.00 | 2.15E-03 | | 0.28 | |
| *POLR2A* | 0.27 | 2.04E-03 | | -0.57 | *ATP5G1* | | 3.92 | 2.36E-03 | | 0.26 | |
| *POLR2E* | 0.2 | 4.81E-03 | |  | *COX8A* | | 3.16 | 3.77E-03 | |  | |
| *POLR3F* | 3.83 | 6.34E-03 | |  | *COX6B1* | | 2.96 | 4.48E-03 | |  | |
| *DUT* | 3.13 | 2.29E-02 | |  | *COX7A2* | | 2.41 | 7.34E-03 | |  | |
| *POLR2H* | 2.16 | 2.31E-02 | | 0.28 | *UQCRB* | | 3.56 | 9.75E-03 | |  | |
| *CTPS2* | 2.62 | 2.39E-02 | |  | *UQCRH* | | 1.94 | 1.01E-02 | | 0.35 | |
| *POLR1A* | 2.62 | 2.91E-02 | |  | *PPA1* | | 1.95 | 1.17E-02 | |  | |
| *POLR2B* | 2.61 | 3.21E-02 | |  | *NDUFC2* | | 2.35 | 1.23E-02 | |  | |
| *NT5C1B* | 5.94 | 3.22E-02 | |  | *LHPP* | | 0.21 | 1.28E-02 | |  | |
| *TXNRD1* | 2.56 | 3.96E-02 | |  | *NDUFB2* | | 3.66 | 1.38E-02 | |  | |
| *UMPS* | 2.65 | 4.30E-02 | |  | *NDUFS6* | | 3.59 | 1.46E-02 | |  | |
| *UCKL1* | 3.25 | 4.50E-02 | |  | *ATP5C1* | | 2.4 | 1.55E-02 | |  | |
| *CANT1* |  |  | | -0.04 | *NDUFAB1* | | 1.88 | 1.59E-02 | |  | |
| *ENTPD1* |  |  | | 0.28 | *PPA2* | | 3.06 | 2.15E-02 | |  | |
| *ENTPD5* |  |  | | -0.02 | *UQCRC2* | | 2.91 | 2.34E-02 | |  | |
| *PRIM2* |  |  | | -0.08 | *COX7B* | | 1.46 | 3.66E-02 | |  | |
|  |  |  | |  | *COX5A* | | 1.97 | 3.99E-02 | |  | |
|  |  |  | |  | *ATP6V0B* | | 2.49 | 4.03E-02 | |  | |
|  |  |  | |  | *NDUFB9* | | 1.99 | 4.20E-02 | |  | |
|  |  |  | |  | *NDUFB6* | | 2.13 | 4.21E-02 | |  | |
|  |  |  | |  | *ATP5J2* | | 1.83 | 4.24E-02 | |  | |
|  |  |  | |  | *ATP4B* | | 3.94 | 4.56E-02 | |  | |
|  |  |  | |  | *ATP5A1* | |  |  | | -0.85 | |
|  |  |  | |  | *ATP6V0A2* | |  |  | | -0.45 | |
|  |  |  | |  | *NDUFS5* | |  |  | | 0.12 | |
|  |  |  | |  | *SDHD* | |  |  | | -0.01 | |

**Table S5 Global test p-value for pathway-level associations with lethal prostate cancer exploring potential effect modifiers**

| **Pathway (n genes)** | **Accounting for Gleason grade in the alternate model^a^ (NL=291/L=113)** | **Among low stage tumors (T1/T2 N0/Nx M0/Mx) (NL=271/L=79)** | **Among high stage tumors (T3 N0/Nx M0/Mx and T4/N1/M1) (NL=17/L=30)** | **Among men BMI<25 at diagnosis (NL=145/L=52)** | **Among men BMI >25 at diagnosis**  **(NL=146/L=61)** |
| --- | --- | --- | --- | --- | --- |
| **Fatty acid metabolism (n=39)** | 3.7x10^-3^ | 1.8x10^-4^ | 0.44 | 0.02 | 6.1x10^-4^ |
| **Glycolysis/Gluconeogenesis (n=62)** | 5.6x10^-6^ | 1.2x10^-8^ | 0.61 | 6.6x10^-6^ | 4.9x10^-6^ |
| **Pentose phosphate (n=27)** | 0.01 | 8.4x10^-4^ | 0.66 | 0.05 | 1.1x10^-3^ |
| **Purine metabolism (n=157)** | 1.3x10^-4^ | 1.7x10^-6^ | 0.10 | 1.5x10^-3^ | 1.9x10^-7^ |
| **Pyrimidine metabolism (n=96)** | 3.5 x10^-4^ | 1.3x10^-5^ | 0.19 | 2.9x10^-3^ | 9.1x10^-6^ |
| **Oxidative phosphorylation (n=123)** | 1.7x10^-3^ | 3.0x10^-5^ | 0.69 | 6.3x10^-3^ | 2.5x10^-4^ |
| **TCA (n=32)** | 0.42 | 0.18 | 0.66 | 0.26 | 0.33 |

*All models compare the full gene model to an alternate model adjusting for age at diagnosis , cohort, year of diagnosis and BMI at diagnosis and ^a^Gleason Grade (categorized as 2-6, 3+4, 4+3, ≥8)*

*T=Tumor, N=Normal, NL=Non-lethal, L= Lethal*

**Figure S1 Strength (log odds ratios) and significance (log p-values) of individual gene associations with Gleason grade ≥8 (n=106) versus Gleason grade 2-7 (n=298) tumors across the seven pathways**


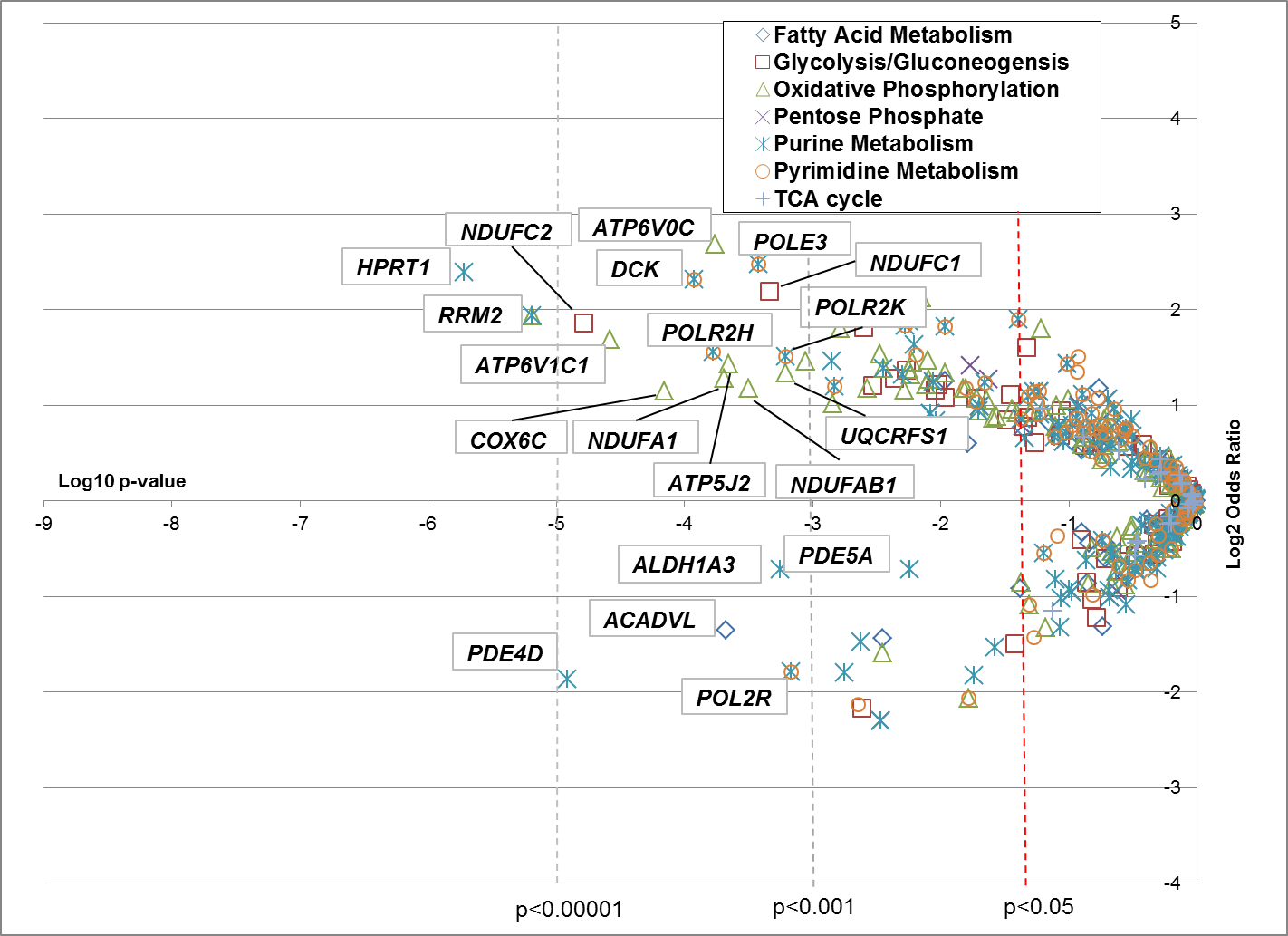


*Log odds ratios computed using a logistic regression model adjusting for age at diagnosis, cohort, year of diagnosis and BMI at diagnosis*

**Figure S2 Strength (log odds ratios) and significance (log p-values) of individual gene associations with lethal prostate cancer among the seven metabolic pathways after additional adjustment for Gleason grade**


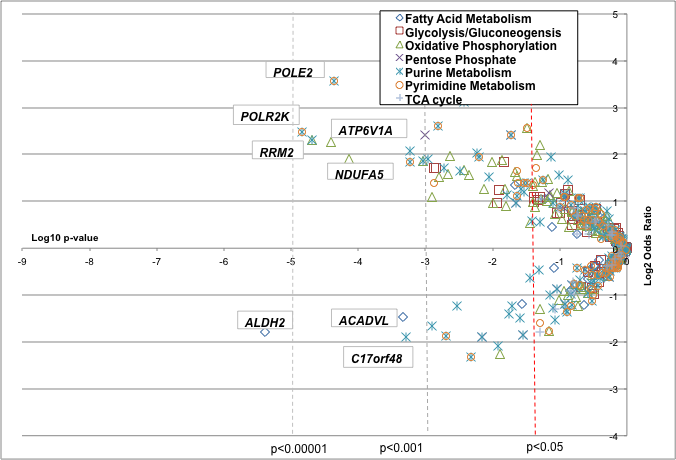


*log odds ratios computed using a logistic regression model adjusting for age, cohort, year of diagnosis, BMI at diagnosis and Gleason grade (categorized as 2-6, 3+4, 4+3, ≥8)*
